# *In-situ* Observation of Size and Irradiation Effects on Thermoelectric Properties of Bi-Sb-Te Nanowire in FIB Trimming

**DOI:** 10.1038/srep23672

**Published:** 2016-03-31

**Authors:** Chia-Hua Chien, Ping-Chung Lee, Wei-Han Tsai, Chien-Hung Lin, Chih-Hao Lee, Yang-Yuan Chen

**Affiliations:** 1 Department of Engineering and System Science, National Tsing Hua University, Hsinchu 300, Taiwan; 2 Nano Science and Technology Program, Taiwan International Graduate Program, Academia Sinica and National Tsing Hua University; 3 Institute of Physics, Academia Sinica, Taipei 11529, Taiwan

## Abstract

In this report, the thermoelectric properties of a Bi_0.8_Sb_1.2_Te_2.9_ nanowire (NW) were *in-situ* studied as it was trimmed from 750 down to 490 and 285 nm in diameter by a focused ion beam. While electrical and thermal conductivities both indubitably decrease with the diameter reduction, the two physical properties clearly exhibit different diameter dependent behaviors. For 750 and 490 nm NWs, much lower thermal conductivities (0.72 and 0.69 W/m-K respectively) were observed as compared with the theoretical prediction of Callaway model. The consequence indicates that in addition to the size effect, extra phonon scattering of defects created by Ga ion irradiation was attributed to the reduction of thermal conductivities. As the NW was further trimmed down to 285 nm, both the electrical and thermal conductivities exhibited a dramatic reduction which was ascribed to the formation of amorphous structure due to Ga ion irradiation. The size dependence of Seebeck coefficient and figure of merit (ZT) show the maximum at 750 nm, then decrease linearly with size decrease. The study not only provides the thoroughly understanding of the size and defect effects on the thermoelectric properties but also proposes a possible method to manipulate the thermal conductivity of NWs via ion irradiation.

Size effects on thermal and transport properties have been extensively studied on the thickness dependence in two dimensional systems, however there are limited literatures and reports on the 1D nanowires (NWs). Due to the vast surfaces and easier oxidation in air, the intrinsic properties of the NWs are hardly examined. To overcome these problems, an *in-situ* measurement method to simultaneously investigate the variation of thermal and transport properties of a single NW has been designed while the NW was trimmed down from 750 to 285 nm by a focused ion beam (FIB). Although both electrical and thermal conductivities indubitably decrease with the diameter reduction is expected due to the confinement effect, their detailed mechanism of phonon and electron scatterings has not been fully understood. If this mechanism can be understood further, then the material engineering for practical applications can be realized. One typical example of these is the searching of high performance thermoelectric materials for waste heat recovery. Owing to the energy crisis and global warming, reducing the energy consumption by energy recycling is demanded. The efficiency of TE material is deduced by the dimensionless figure of merit (ZT), written by


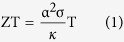


where α, σ, κ, and T represent the Seebeck coefficient, electrical conductivity, thermal conductivity, and absolute temperature respectively. Obviously electrical and thermal conductivities are two crucial parameters to be tuned for the enhancement of the figure of merit (ZT). Low-dimensional nanostructures are the potential candidates to achieve the goal in terms of their vast phonon-interface scatterings^1^. Especially for the one-dimensional materials, such as nanobelts^2^,^3^, nanotubes^4^,^5^,^6^ , and NWs^7^,^8^, as the diameter is comparable to their phonon mean free path, the reduction of thermal conductivity can be achieved. Dresselhaus *et al*.^9^ theoretically showed that for one-dimensional TE material, the ZT enhancement is mainly attributed to thermal conductivity reduction. This motivates our researches on the thermoelectricity in one-dimensional NWs. C.L. Chen *et al*.^10^ showed that 120 nm n-type Bi_2_Te_3_ NWs array grown by electrodeposition displayed a significant low thermal conductivity 0.75 W/m-K at 300 K, which is about only one-third of its bulk value. A similar result also reported in p-type Bi_0.5_Sb_1.5_Te_3_ nanowires of 60–280 nm in diameter^11^. Due to the superior thermoelectric performance of Bismuth-Antimony-Telluride (BST) materials near room temperature^12^, Bi_2-x_Sb_x_Te_3-y_ nanowires are selected as the specimens in the work.

In this work, the highly crystalline BST NWs were grown directly from Bi_0.5_Sb_1.5_Te_3_ thin film by thermal annealing^13^ without using any precursor or catalyst to avoid any possible parasitic influence in NWs. The selected BST NW was transferred and suspended on the measurement platform^14^, and then mounted in a FIB (DBFIB-SEM, FEI NOVA-600) chamber for preventing oxidation, contamination and environmental influence. By the design, the diameter dependence of electrical conductivity, thermal conductivity, Seebeck coefficient and heat capacity can be simultaneously characterized through FIB trimming processes. Figure 1b shows the top view of the measurement platform and the electrical leading wires. The SEM images of NWs with 750, 490, and 285 nm NWs are shown in Fig. 1c–e, respectively. The chemical stoichiometric composition analysis by SEM-EDS can also be performed *in-situ* in the FIB chamber.

## Results

### Structure and chemical composition

The selected area electron diffraction (SAED) and the high resolution transmission electron microscopy (HRTEM) of the pristine 750 nm NW and trimmed 285 nm NW are shown in Fig. 2a,b respectively. The single crystallinity and hexagonal structure with the [110] growth direction in the axis of NW are clearly shown in 750 nm NW. The results are in consistent with that of Bi_2_Te_3_ NW^13^ grown by the same method. The lattice fringes and the inter-planar spacing d = 0.21 nm of {110} planes are also shown in the inset to Fig. 2a. After the trimming processes, the SAED pattern of 285 nm NW (Fig. 2b) shows amorphous-like characteristics, which is visibly revealed by the diffraction rings and spots. In addition to the diffraction rings of BST {110} plane, the Ga {015} and {232} planes are also shown in the inset of Fig. 2b, indicating the Ga ions were implanted in the 285 nm NW. Meanwhile, the amorphous-like structure formed by the dynamic annealing^15^ of FIB trimming processes is directly confirmed by the HRTEM image in the inset of Fig. 2b as well.

The *in-situ* characterization of stoichiometric composition of NW was performed by SEM-EDS and listed in Table 1. The amount of Ga ions implanted in NWs is represented by the atomic percentage. The reference bulk with same growth direction and composition was prepared and measured for comparison purpose^16^. The composition analysis showed that the amount of tellurium in NWs was reduced after FIB trimming process due to the decomposition and vaporization of Te during the dynamic annealing. It is noticed that, since the probe depth of SEM-EDX is limited in the range of about 5–50 nm, the composition result is more like of surface layer.

### Electrical conductivity

In order to monitor the condition of NWs, the electrical resistance of BST NW was simultaneously measured through the whole FIB trimming processes. The time dependence of electrical resistance is shown in Fig. 3. Basically, the resistance increase as expected when the diameter of NW decreases in time. The resistance increased from 250 to 1000 and 20 kΩ, as the NW was trimmed down from 750 to 490 and 285 nm respectively. Examining the intrinsic electrical conductivity, the pristine 750 nm and the trimmed 490 nm NWs show similar value as that of the bulk (Fig. 4a), indicating the electron carriers are not much influenced by the geometric size and ion irradiation. A slight increase of 9% electrical conductivity observed in 490 nm NW is attributed to extra Ga implanted by irradiation was confirmed by SEM-EDX^17^,^18^. As the wire was further trimmed down to 285 nm, a 74% rapid drop from 0.086 to 0.022 S/μm was observed. This consequence is consistent with the amorphous-like structure discussed above (the inset to Fig. 2b).

The temperature coefficient of resistance (TCR) calculated from R(T) for 300–310 K first slowly decreases from 5.2 × 10^−3^ to 3.4 × 10^−3^ K^−1^ and then to 2.7 × 10^−3^ K^−1^ for the bulk, 750 and 490 nm NW respectively. It then drastically decreases to 0.7 × 10^−3^ K^−1^ for 285 nm NW (Table 1). This is another evidence of crystalline to amorphous-like structure transition as the NW was trimmed down from 490 to 285 nm. A similar result has been reported by S.Y. Glazkov *et al*.^19^ in which the additional defects and poor crystallinity have also been observed.

### Thermal conductivity

The diameter-dependent thermal conductivities of NW were carried out by a self-heating 3ω technique^11^,^20^,^21^ which has been commonly employed to measure the thermal dynamic properties of NWs. In Fig. 4a, a drastic decrease of thermal conductivity in 750 and 490 nm NWs was shown as compared with the bulk, implying the emergence of phonon scatterings of size confinement in the diameter region. Furthermore, the experimental results are ~30% smaller than the theoretical values calculated by Callaway model and Wiedemann-Franz law, (open circles in Fig. 4a) with the same parameters of point defect as BST bulk^22^. The deviation is ascribed to the additional vacancies and disorders created during ion irradiation process. As the NW was further trimmed down to 285 nm, the thermal conductivity exhibited a 74% dramatic drop due to the substantial formation of amorphous-like structure which was approved by HRTEM images (Fig. 2b).

The size dependence of thermal conductivity can be understood by quantitative analysis. The total thermal conductivity *κ* is contributed both by *κ*_e_ of electronic carries and *κ*_p_ of lattice phonons. Since the electronic part can be estimated from electrical conductivity through the Wiedemann-Franz law, *κ*_*e*_ = LσT, where L is Lorenz number. Thus the net contribution of lattice phonons can be obtained from total thermal conductivity by subtracting electronic portion. The Lorenz number for the bulk is 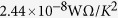 based on the classical free-electron model. It is taken as 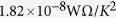 for NW based on the size effect and interface scattering of electrons in NWs^23^. According to the Callaway model^24^ the lattice thermal conductivity is expressed as


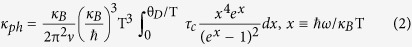


where υ is the average sound velocity, θ_D_ is the Debye temperature and *x* is the dimensionless parameter including reduced Plank constant ħ, angular frequency ω and Boltzmann constant *k*_*B*_. The combined relaxation rate τ_c_^−1^ can be obtained by Matthiessen’s rule from three sources which are written as


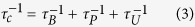


where 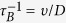; *υ* is the phonon group velocity and D is the diameter of NW. 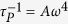; A is the point defect scattering parameter. 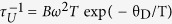; B is the umklapp scattering parameter. The Callaway model has been commonly used for lattice thermal conductivity simulation and been well fitted to bismuth -telluride based material^25^.

In Fig. 5a, the phonon thermal conductivities of bulk and reference data (represented by star symbols)^11^ are well fitted to the Callaway model^24^ . The experimental data (solid symbols) of trimmed BST NW are much lower than the simulation curve due to the vacancies and disorders created by ion irradiation. In the plot, the effective diameter of 285 nm NW specimen is represented as 10 nm which is the average grain size of the specimen (Fig. 2a).

In order to study the temperature dependence of phonon thermal conductivity, a 280 nm pristine Bi_0.75_Sb_1.25_Te_2.86_ NW was prepared. Figure 5b shows the lattice thermal conductivity of the NW and the simulation curve of the BST bulk from Fig. 5a. The temperature dependence of phonon thermal conductivity of NW specimen shows the shifting of phonon drag to higher temperature (~80 K) and the suppression of thermal conductivity, these are the features of the enhanced phonon-boundary scattering^7^,^26^.

### Seebeck coefficient and Figure of merit (ZT)

The Seebeck coefficient data of the bulk and NWs are shown in Fig. 4b exhibiting p-type behavior. The size dependence of Seebeck coefficient and figure of merit (ZT) show the maximum at 750 nm, then decrease linearly with size decrease. The uncertainty in ZT determination is about 20% based on the error analysis from electrical conductivity, thermal conductivity and Seebeck coefficient measurements. The Seebeck coefficient decreases from 167 to 102 μV/K as NW trimmed down from 750 nm to 285 nm. This 40% decrease may be due to ion implantation and additional grain boundary scattering^27^. In Fig. 4b the size dependence of ZT shows analog trend as that of Seebeck coefficient, thus, the lower figure of merit of 490 and 285 nm NWs is mainly due to their smaller Seebeck coefficient.

## Discussion

The diameter dependence of thermoelectric properties of single Bi_2-x_Sb_x_Te_3-y_ NW has been *in-situ* studied in FIB trimming. As the diameter was reduced from the bulk to 750 and 490 nm, a drastic decrease of thermal conductivity but electrical conductivity was distinctly seen.

The consequence implies the size and defect effects have more influence on phonon scattering than that of electron carriers in this diameter region. When the NW was further trimmed to 285 nm, both the electrical and thermal conductivities started a dramatic drop due to the formation of a large number of defects and the amorphous-like structure by severe Ga ion irradiation which was confirmed by HRTEM images in FIB trimming. As compared with the bulk counterpart, the novel physical properties of thermal/electrical conductivity and Seebeck coefficient in NWs had been revealed. In addition to the renowned size effect, the ion irradiation induced amorphous-like structure also plays a significant role in thermal conductivity reduction. The study not only provides the thoroughly understanding of the size and ion irradiation effects on the electrical and thermal transport properties but also provides a possible method to manipulate the physical properties in nanostructures for the extensive applications in engineering high ZT thermoelectric materials.

## Methods

### Sample preparation

**Figure 1:**
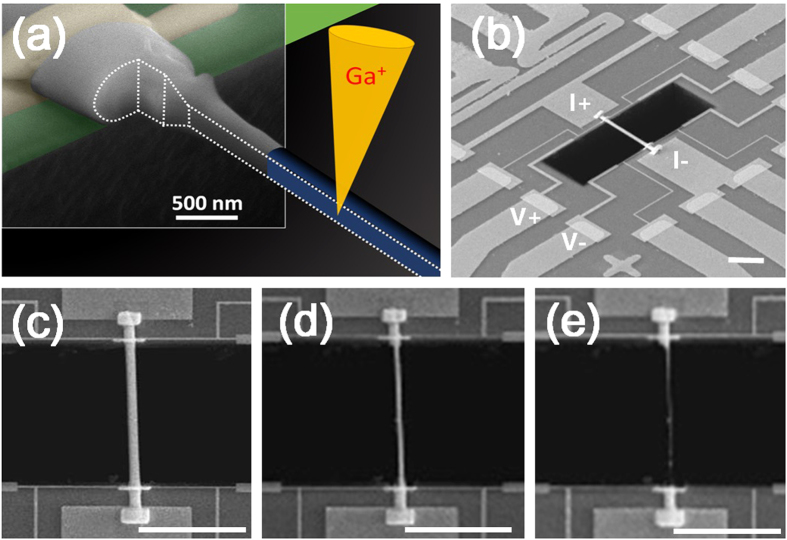
The FIB trimming processes, measurement platform, and trimmed BST NWs. (**a**) The semi-schematic diagram of the suspended NW on the measurement platform and the trimming processes of FIB. The upper left shows the real image of NW taken by SEM. The gold pads are the electrical leading wires for experimental measurements and the dash line indicating the cutting edge of the trimmed BST NW. (**b**) The SEM image of the suspended BST NW and the electrodes. (**c**–**e**) The SEM images of pristine and trimmed BST NW with sizes of 750, 490 and 285 nm, respectively. The scale bars in the figures are 10 μm.

**Figure 2:**
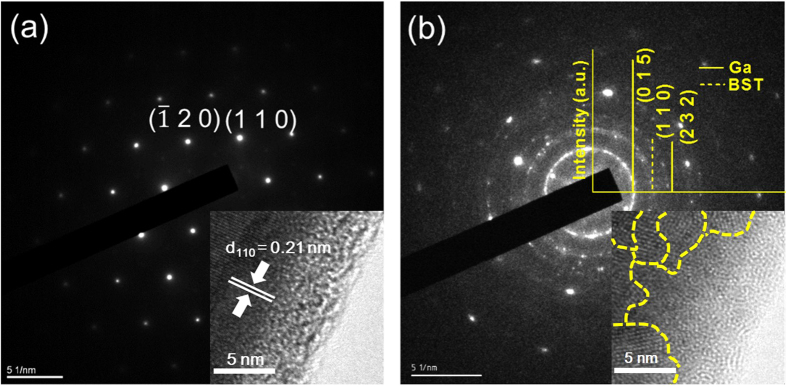
Electron diffraction patterns of the pristine and trimmed BST NWs. (**a**) The SAED pattern of pristine NW with diameter 750 nm, and the inset shows the HRTEM picture with d_110_ = 0.21 nm. (**b**) The SAED pattern of the trimmed 285 nm NW. The upper right corner is the correlated diffraction pattern collected from SAED result, which identifies the BST (dot line) and Ga (solid line) are both presented in the specimen. Inset shows the HRTEM image of polycrystalline lattice structure, the dash lines are eye guides for the lattice discontinuity. All diffraction patterns are taken along [001] zone axis.

**Figure 3:**
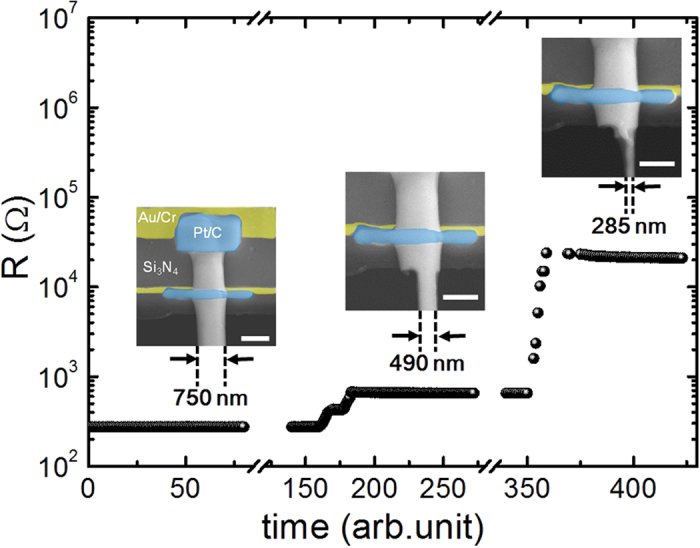
Electrical resistance changes with time as the NW is trimmed to smaller diameter. Insets show the SEM images of NW near the top electrode which indicate three steps in trimming processes with diameter 750, 490 and 285 nm respectively. The pre-deposited Au/Cr electrodes with gold color are used for four-probe resistance measurement, and Pt/C contact pads (blue color) deposited by FIB are used to amend electrical contacts. The scale bars in the insets are 1 μm.

**Figure 4:**
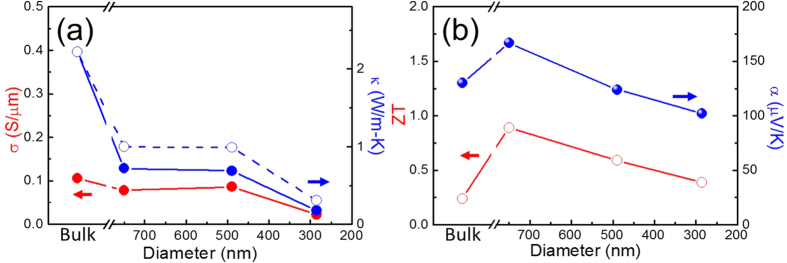
The diameter dependence of thermoelectric properties. (**a**) The diameter dependence of electrical and thermal conductivities (solid circles). The data of thermal conductivity calculated by Callaway model and Wiedemann-Franz law are presented by open circles. (**b**) The size dependence of Seebeck coefficient and figure of merit (ZT) are represented by solid circles and open circles respectively.

**Figure 5:**
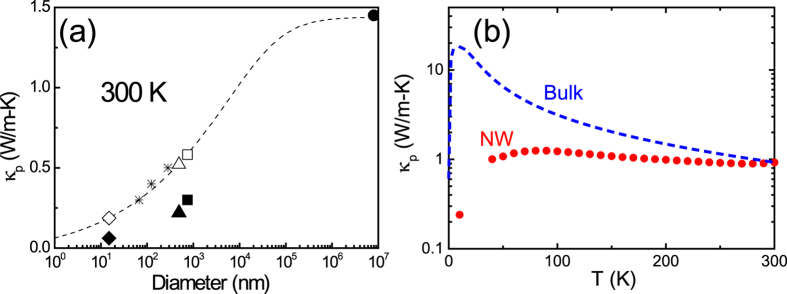
The experimental phonon thermal conductivity and Callaway model simulation. (**a**) The solid circle, cube, triangle and diamond represent the experimental data of the bulk and NWs. The open symbols show the simulated phonon thermal conductivity without Ga ion irradiation. The star symbols represent the data from Liang Li *et al*.^11^ measured by the same technique, and the dash line shows the Callaway model simulation curve. (**b**) The experimental lattice thermal conductivity data of pristine 280 nm NW with no ion irradiation (solid circle) and the simulation curve of BST bulk (dash line).

The thermal annealing method^13^ which is a convenient and clean way to grow the NWs with high aspect ratio was applied to synthesize the single crystalline BST NWs. The Bi_0.5_Sb_1.5_Te_3_ films were deposited on SiO_2_/Si substrates by pulse laser deposition under high vacuum (~2 × 10^−6^ torr). Then, the as grown BST films were sealed in the quartz tube under 5 × 10^−6^ torr and annealed at 340 °C for 5 days. According to the thermal expansion coefficient difference between SiO_2_ (~2.4 × 10^−6^/°C)/Si (~0.5 × 10^−6^/°C) substrate and BST (~13.4 × 10^−6^/°C) thin film, the highly crystalline NWs with diameter range of 200 to 1000 nm were obtained. Figure 2a shows the HR-TEM image and the SAED pattern in [001] zone axis of pristine BST 750 nm NW. The results show a single-crystalline structure and [110] growth direction in NW. The tungsten probes with tip diameter of 100 nm were used to transfer the selected NW from the as-grown sample onto the open window in the center of measurement platform for thermoelectric properties measurements (Fig. 1). The Pt/C pads post deposited by FIB (inset of Fig. 3) to ensure the solid thermal and electrical contact between NW and electrodes. To study the size effect of NW sample, the focused Ga ion beam (dose is ~10^18^ cm^−2^ at 30 kV with spot size 12.5 nm) was used to trim the pristine NW from 750 nm to 490 and 285 nm sequentially. Since the cross section of NW is not completely circular, thus the corresponding diameter of NW was derived from the formula πr^2^ = A, where 2r is the diameter, and A is the cross section area.

### Electrical and Seebeck coefficient characterization

The electrical conductivity was measured by standard four- probes method to avoid the influence from leading wire and contact resistance. The applied current was also tested and monitored to ensure no noticeable joule heat in the resistance measurements. The sample holder was designed for easy sample mounting and temperature control in the range of 300–320 K. The AC current source (Keithley, 6221) and the lock-in amplifier (Signal recovery, 7265) were then connected to the template through electrical feedthrough for data characterization. The alternating Seebeck coefficient measurement technique^14^,^28^ was applied to measure the Seebeck coefficient of trimmed BST NWs (see Supplementary Fig. S2). The measurement errors of electrical conductivity and Seebeck coefficient are estimated ~5%.

### Thermal conductivity and heat capacity characterization

The self-heating 3ω method has been extensively used to characterize the thermal conductivity of 1D wires^29^,^30^ owing to its short thermal equilibrium time and high accuracy^20^. The aspect ratio of pristine BST NW was larger than 10 which meet the boundary condition of one-dimensional like suspended wire in 3ω self-heating method. To avoid the convention heat loss, these measurements were performed under the vacuum of 5 × 10^−5^ torr in FIB chamber. The thermal conductivity (κ) was calculated from the third harmonic voltage signal (V_3ω_) through the formula listed below


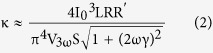


where R and R′ are the resistance and its derivative at the corresponding temperature. L and S are the length and cross section of the NW. The alternating current I = I_0_sinωt was applied to generate appropriate temperature fluctuation on the NW for measurement purpose. γ ≡ L^2^/π^2^α, where α is thermal diffusivity, represents the characteristic thermal time constant of the NW. To verify the reliability of measurement results, three crucial relations among V_3ω_, I, and ω: (1) V_3ω_ ∝ I^3^ (2)
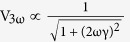
and (3) tan*φ* ∝ ω had been carefully measured and confirmed^20^ (see Supplementary Fig. S1).

The cross-section area of trimmed NWs is critical in the derivation of thermal conductivity and electrical conductivity. In general, the diameter of sample was directly measured by SEM image, however the uncertainty caused by the variation of electromagnetic lens^31^ could influence the accuracy of the measurements. According to the Dulong and Petit’s law, the heat capacitance at high temperatures is related to the atomic weight of the sample. Therefore, the diameter and cross-section area of the specimens were double checked by the heat capacity measurement (*C*) obtained from 3ω method. Experimental results from these two methods are in consistent with each other and the accuracy of the electrical and thermal conductivities are insured. The measurement errors of thermal conductivity are mainly from the uncertainty of third harmonic voltage signal (V_3ω_) which is estimated to be ~5–10%.

## Additional Information

**How to cite this article**: Chien, C.-H. *et al*. *In-situ* Observation of Size and Irradiation Effects on Thermoelectric Properties of Bi-Sb-Te Nanowire in FIB Trimming. *Sci. Rep*. **6**, 23672; doi: 10.1038/srep23672 (2016).

## Supplementary Material

Supplementary Information

## Figures and Tables

**Table 1:** The heat capacity (*C*) by theoretical calculation, electrical thermal conductivity (κ_e_), lattice thermal conductivity (κ_p_), electrical conductivity (σ), temperature coefficient of resistance (TCR) of NW specimens and its bulk counterpart at 300 K.

Diameter (nm)	Bulk	750	490	285
Chemical composition	Bi_0.8_Sb_1.2_Te_2.9_	Bi_0.8_Sb_1.2_Te_2.9_	Bi_0.5_Sb_1.2_Te_2.6_ + 15 at.% Ga	Bi_0.5_Sb_1.2_Te_1.6_ + 33 at.% Ga
*C* (J/g-K)	0.16	0.16	0.15	0.20
κ_e_ (W/m-K)	0.77	0.42	0.47	0.12
κ_p_ (W/m-K)	1.45	0.3	0.22	0.06
σ (S/μm)	0.106	0.078	0.086	0.022
TCR (K^−1^)	5.2 × 10^−3^	3.4 × 10^−3^	2.7 × 10^−3^	0.7 × 10^−3^

Note: The chemical composition of the bulk is nominal, whereas the compositions of NWs are obtained from SEM-EDX.
