# Supplementary material for: In-situ Observation of Size and Irradiation Effects on Thermoelectric Properties of Bi-Sb-Te Nanowire in FIB Trimming
Source: Sci Rep. 2016 Mar 31;6:23672. doi: 10.1038/srep23672 (PMC4814841; doi:10.1038/srep23672)
Supplement: Supplementary Information [file srep23672-s1.pdf]

Supplementary Information

In-situ Observation of Size and Irradiation Effects on Thermoelectric Properties of Bi-Sb-Te Nanowire in FIB Trimming

Chia-Hua Chien[1,2,3], Ping-Chung Lee[3,*], Wei-Han Tsai[3], Chien-Hung Lin[3], Chih-Hao Lee[1], and Yang-Yuan Chen[3,*]

[1]Department of Engineering and System Science, National Tsing Hua University, Hsinchu 300, Taiwan.

[2]Nano Science and Technology Program, Taiwan International Graduate Program, Academia Sinica and National Tsing Hua University.

[3]Institute of Physics, Academia Sinica, Taipei 11529, Taiwan.

[*]Correspondence and requests of materials should be addressed to

iamplex@phys.sinica.edu.tw. , cheny2@phys.sinica.edu.tw.

Thermal conductivity measurement. To check whether each NW satisfies the boundary conditions of self-heating 3ω method, such as the appropriate range of current and frequency, three crucial measurements based on Eq. (2) had been carefully examined. Fig. S1a shows the current dependence of $V_{3\omega}$ at 25.3 Hz (the inset shows the enlarged scale of 285 nm all $V_{3\omega}$ is a linear function of I^3. The phase angle tan φ of $V_{3\omega}$ is a linear dependence of frequency (Fig. S1b). And the frequency dependence of $V_{3\omega}$ is well fitted to function of $\frac{1}{\sqrt{1+(2\omega\gamma)^2}}$ (Fig. S1 c, d, e). The error of 5-10 % from the thermal conductivity measurement is mainly estimated by the standard deviation of curve fitting.

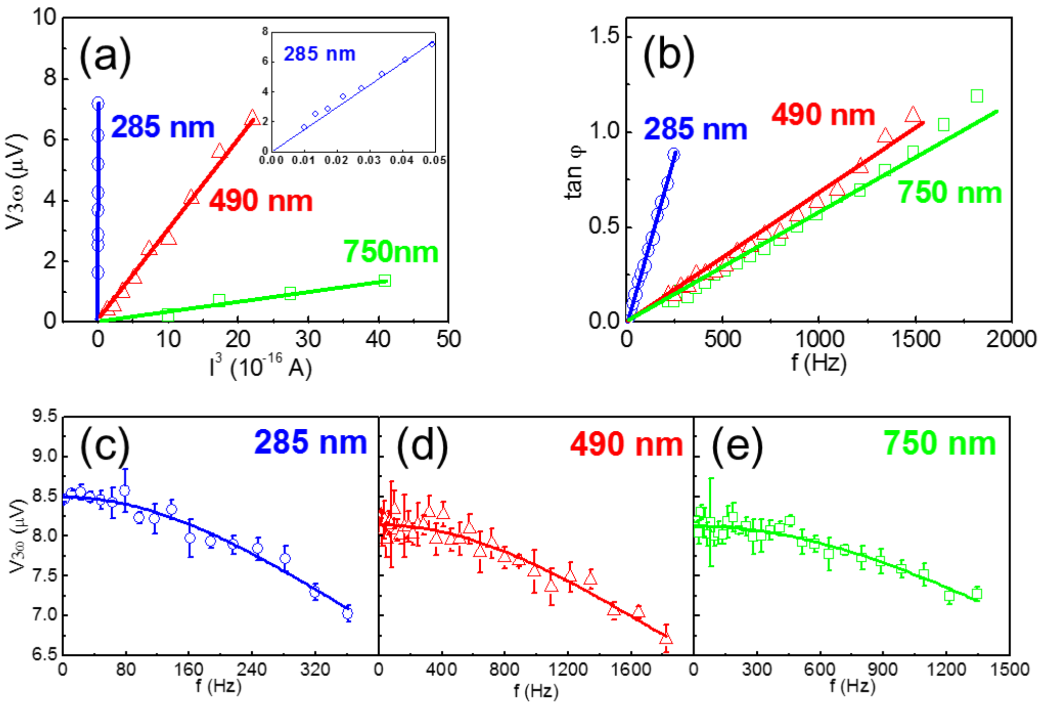

Figure S1. Experimental data of self-heating 3ω method. (a) $V_{3\omega}$ versus I^3, (b) phase angle φ versus frequency, (c), (d), (e) $V_{3\omega}$ versus frequency for three NW

specimens at 300 K. Solid lines and open symbols represent the curve fitting and the measured data, respectively. (750 nm represented by cubes, 490 nm represented by triangles and 285 nm represented by circles)

Seebeck coefficient measurement. The Seebeck coefficient is measured by alternating current technique and calculated from the linear fit of electromotive force (EMF) versus temperature gradient ($T_{hot} - T_{cold}$) (Fig. S2e). Fig. S2a, b, and c show the temperature variation for both hot side and cold side temperature sensors after heating power is turned on. The temperature sensors were carefully calibrated before the measurements (Fig S2d).

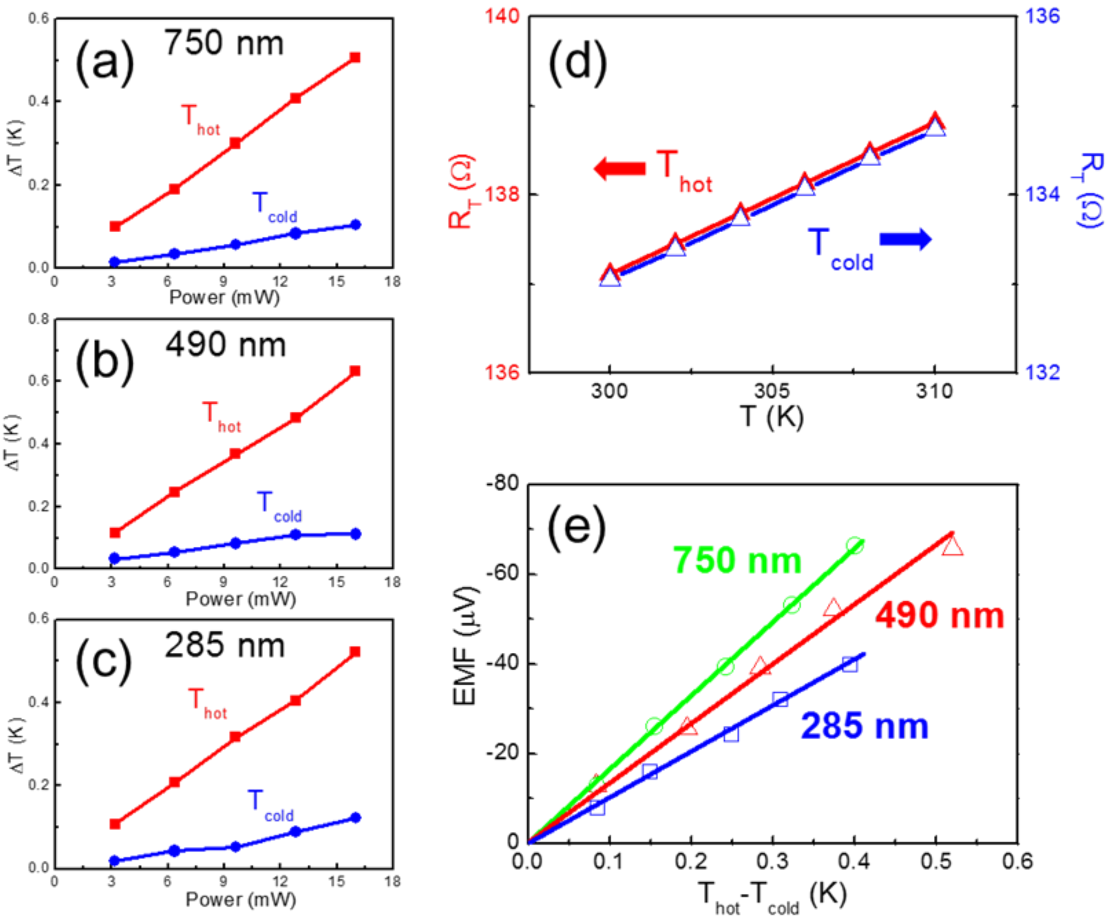

Figure S2. Experimental data of Seebeck coefficient measurement at 300 K. (a) (b) (c) The temperature variation (ΔT) of two temperature sensors, hot side (red cubes) and cold side (blue circles), as a function of the heating power. (d) The resistance calibration of temperature sensors, for hot side (red triangles) and cold side (blue triangles). (e) EMF versus temperature gradient ($T_{hot} – T_{cold}$) for NWs of 750 nm (open circles), 490 nm (open triangles) and 285 nm (open cubes). The measurement errors of Seebeck coefficient are estimated to be ~ 5 %.

Error analysis. About 5 % error from the diameter of NW due to size geometry, especially after trimming, the diameter doesn't appear to be constant over the length of the trimmed NW. Uncertainty of temperature sensor for thermal conductivity and Seebeck coefficient measurements is ~5 %. Finally, the total uncertainty in ZT is estimated ~20 % resulted from the errors mentioned.
